# Supplementary material for: X-ray contrast-adjustable 3D printing for multimodal fusion of microCT and histology
Source: Front Med Technol. 2026 Jan 9;7:1702201. doi: 10.3389/fmedt.2025.1702201 (PMC12827677; doi:10.3389/fmedt.2025.1702201)
Supplement: Supplementary file 1 [file Datasheet1.pdf]

Supplementary Material

1 SUPPLEMENTARY TABLES AND FIGURES

1.1 Figures

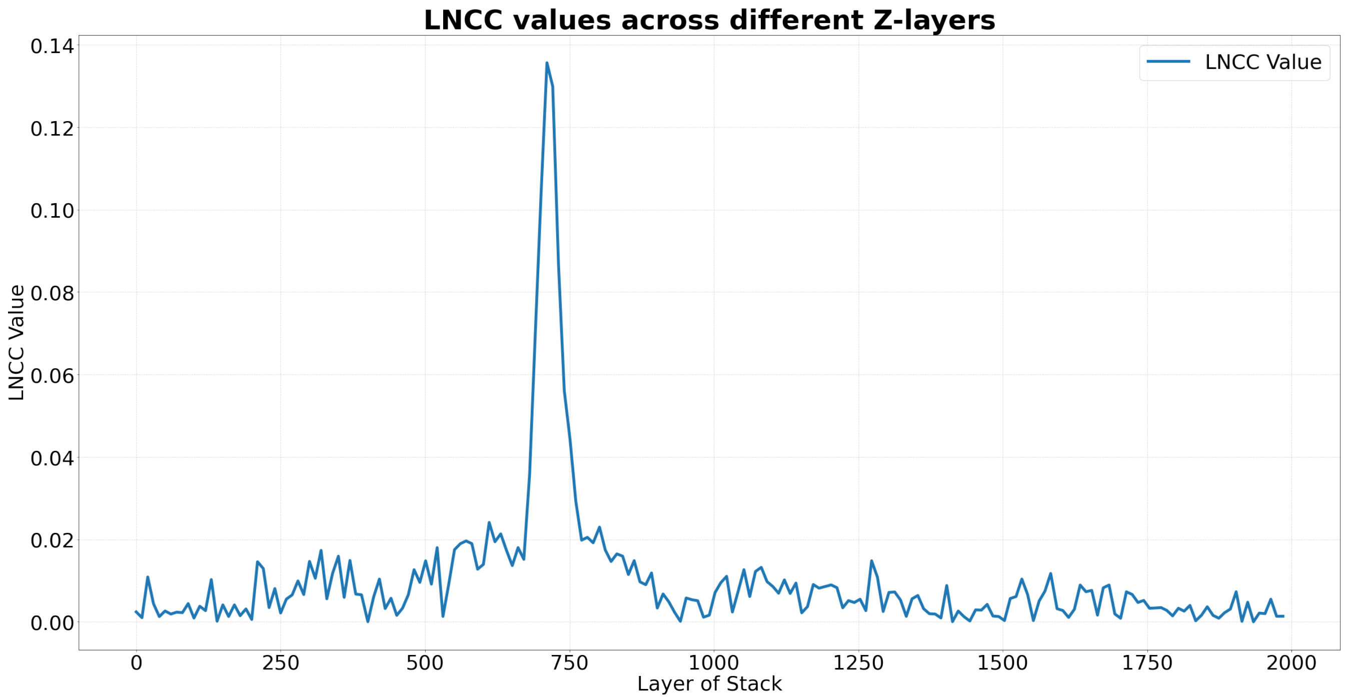

Figure 1a. LNCC scores computed across all layers of the microCT stack.

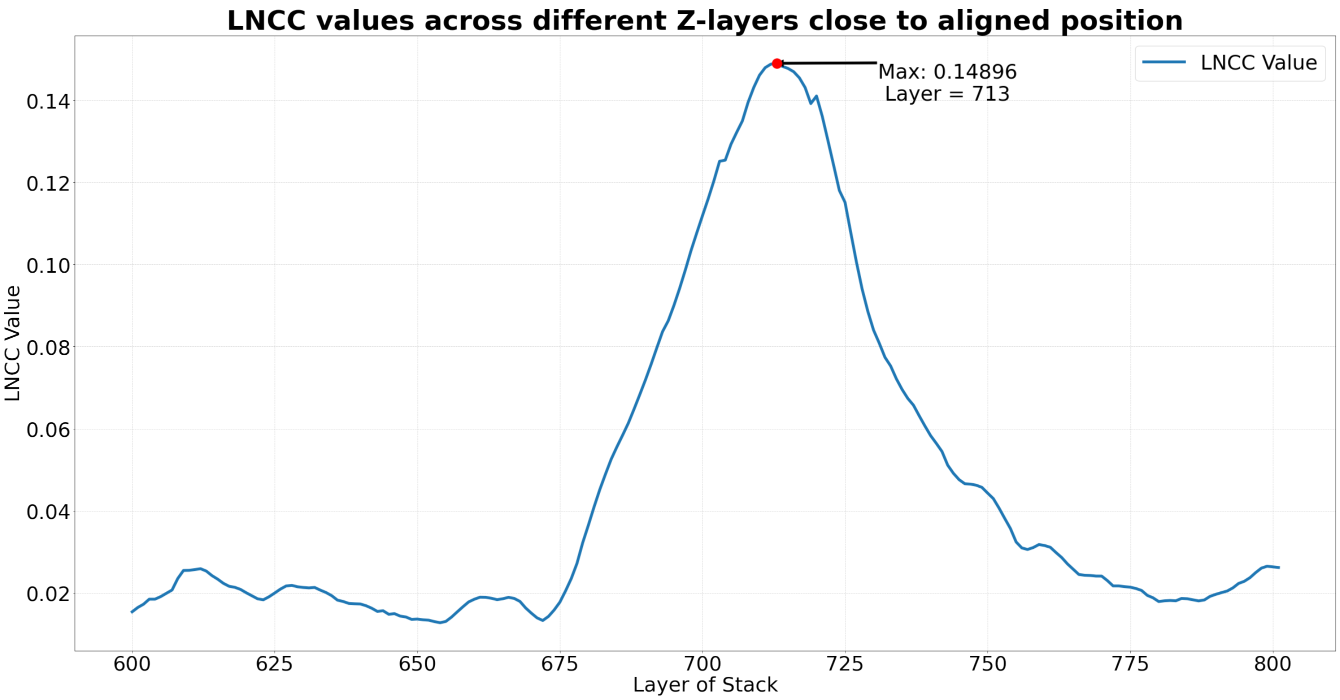

Figure 1b. LNCC scores near the alignment position in layer 713.

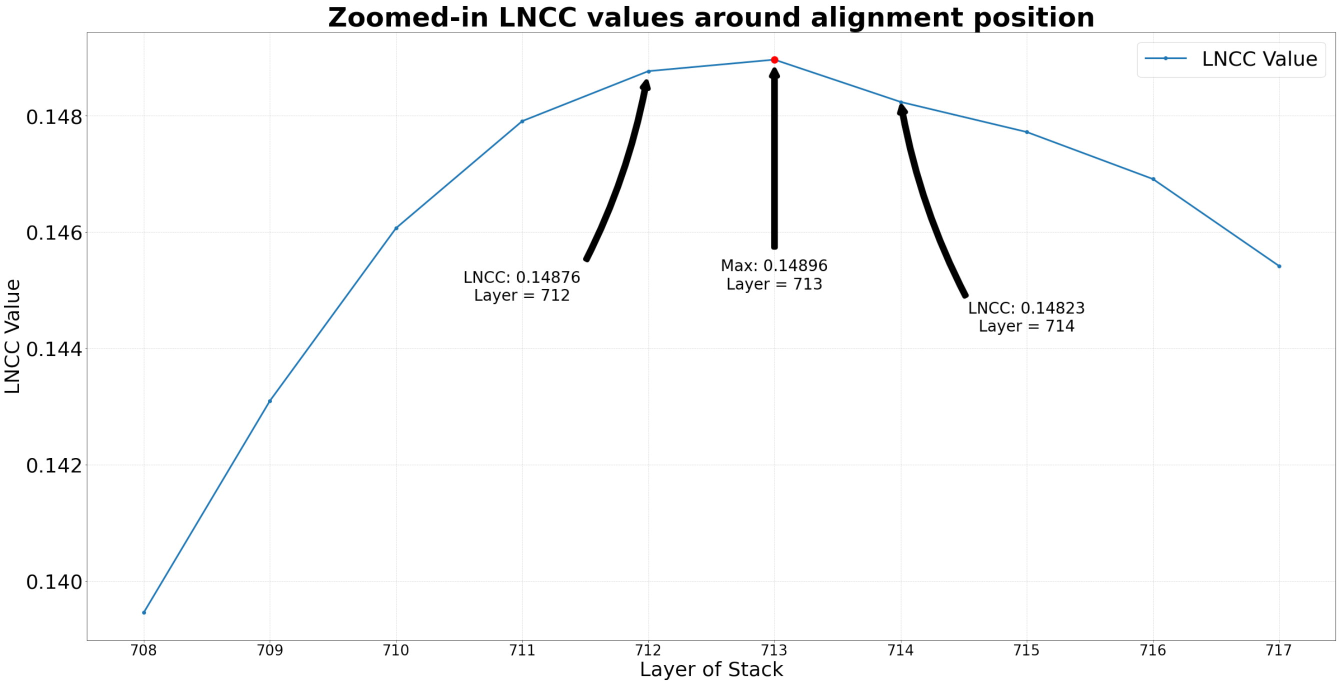

Figure 1c. LNCC values adjacent to the alignment position in layer 713.

Figure 1. Verification of the alignment position based on the conic reference marker. **(A)** LNCC values across the entire stack. **(B)** LNCC values in layers 600–800, highlighting the alignment region. **(C)** LNCC values immediately adjacent to the determined alignment position, which served as the basis for subsequent registration.
